# Supplementary material for: Neuromorphic object localization using resistive memories and ultrasonic transducers
Source: Nat Commun. 2022 Jun 18;13:3506. doi: 10.1038/s41467-022-31157-y (PMC9206646; doi:10.1038/s41467-022-31157-y)
Supplement: Supplementary file 1 — Supplementary Information [file 41467_2022_31157_MOESM1_ESM.pdf]

Supplemental Information

A neuromorphic system based on resistive memories and ultrasonic transducers for object localization

RRAM characterization Resistive Random Access Memories are electronic devices based on the formation and rupture of a conductive filament across an insulator material. Here we consider an oxide-based resistive memory composed of 5 nm of hafnium-dioxide sandwiched between a top and bottom electrode of titanium and titanium-nitride. The cells can be programmed in different conductance states by applying appropriate voltage and current waveforms over the device. The change in the geometry of the filament results in different conductance states in the device. By applying a positive/negative SET/REST voltage between top and bottom electrode, a conductive filament is formed/disrupted, thus increasing/decreasing the conductance. When the filament is formed the cell is in the High Conductive State (HCS), otherwise the cell is in the Low Conductive State (LCS). Resistive memory technologies suffer from conductance variability: if a device is repeatedly cycled under the same programming conditions a conductance distribution emerges. The median conductance and the standard deviation of this distribution are determined by the compliance current (i.e. programming current) applied during the SET operation (I_{CC}). Supplementary Figs. 1a and b show the modulation of the median conductance and standard deviation of the HCS distributions as a function of the compliance current. The measurements have been performed on a 16 kb array of one transistor-one resistor (1T1R) devices. The cells are SET using 15 programming conditions (V_{GS} in $[1.4 - 3.0]V$), resulting in different compliance currents, and $2V$ on the top electrode. The cycle to cycle variability prevents programming of an RRAM cell to a precise conductance value. Numerous techniques have been developed to cope with cycle-to-cycle variability when programming RRAM devices. In [1], a technique has been developed to yield up to 8 different separated levels of conductance from a single RRAM device, by means of an iterative procedure alternating Resets and Set operations. We will refer to it as Smart Programming (SP). Supplementary Fig. 1c shows the difference between a single shot SET operation and the Smart Programming applied to the 16kb array, the latter obtaining a much tighter distribution of devices. The Smart Programming procedure involves recursive programming of a single device, alternating carefully modulated Set and Reset operations, until the conductance of the device falls within a predetermined target range. Nonetheless, this procedure still suffers from relaxation: the conductance distribution obtained just after programming (t=0, black in Supplementary Fig. 1c) rapidly spreads toward both higher and lower values. After 1 hour more than 45% of the programmed devices are out of the target conductance range. A fix for this problem is found in the Relaxation Correction Smart Programming procedure: the flow of this operation is the same as in the standard SP, but a waiting time of 5 s is added between each re-programming operation. Cells that suffer from conductance instability during the waiting period are rescheduled to the next programming iteration, allowing the algorithm to take into account and correct the conductance relaxation. Supplementary Fig. 1d shows the difference between Standard SP (blue) and Relaxation Correction SP (green) on 1096 devices programmed with the same target conductance interval. The black line represents the tight conductance distribution right after the last programming operation (t=0), and the colored ones are measured after 1 hour. Relaxation Correction SP greatly reduces the temporal variability. Supplementary Fig. 1e visualizes the same data plotting the difference of the conductance after 1h with that at $t = 0s$ ($G_{1h} - G_{0s}$). This highlights how single devices were changing their conductance over the course of 1 hour. The green distribution is much tighter that the blue one, meaning that the Relaxation Correction procedure effectively reduced the temporal instability.

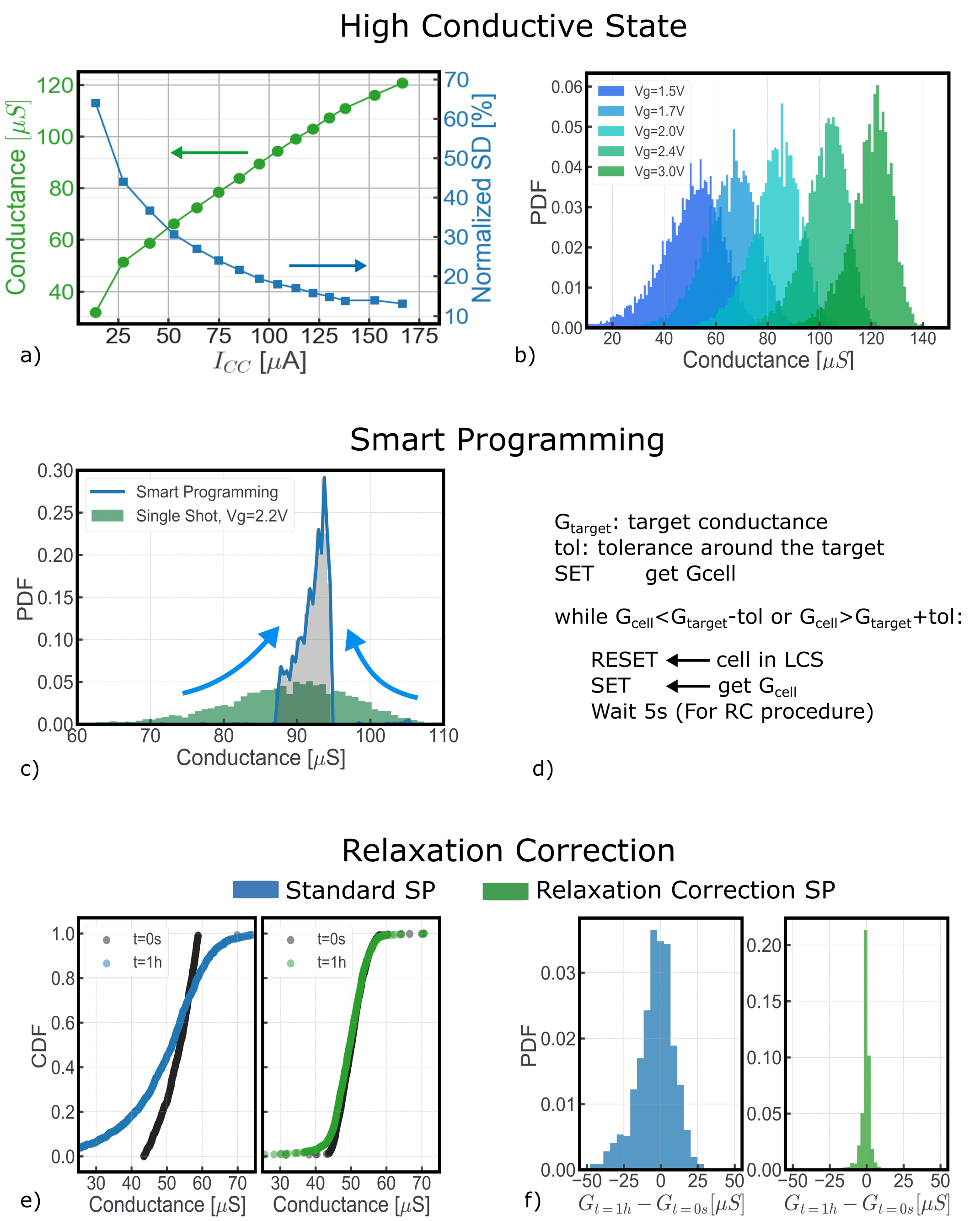

Supplementary Figure 1: Characterization of the RRAM devices from a 16kb array. a) HCS conductance and Normalized Standard Deviation as a function of the compliance current. b) Visualization of some distribution of HCS states with different Compliance current. c) Smart Programming operation solving the problem of variability of the conductive state. d) Description of the recursive programming operation called Smart Programming, with a hint of the Relaxation Correction procedure. e, f) Effect of the Relaxation Correction on the SP procedure. Adding a 5s waiting time between programming iterations reduced the effect of Relaxation and Retention. Left, difference between Standard SP (blue) and RCSP (green) on 1096 devices measured after 1h. Right, distribution of the conductance difference between those measured after programming ($G_{t=0s}$) and after 1 hour ($G_{t=1h}$)

Variability analysis of neuron and synapse analog circuits The two main components of our analog neuromorphic circuits are LIF neurons (Supplementary Fig 2a) and DPI synapses (Supplementary Fig 2b). Both of the circuits are constituted of an input differential pair (DPI) and accumulate charge into a capacitor. The DPI synapse outputs a current corresponding to the first order low-pass-filtered version of the input pulses it receives. For the neuron, when the charge at the capacitor overcomes a predetermined threshold, an output spike is produced and the capacitor's voltage is reset to 0 V by a feedback mechanism. Both circuits are characterized by a time constant given by the rate of discharging of their capacitors and an input gain, which

evaluates the amount of charge accumulated by the capacitor in response to an input pulse. As discussed in the main text, analog neuromorphic circuits suffer from variability, due to imperfections of the CMOS transistors utilized at the sub-threshold regime. This affects the leakage time constant and the input gain. We performed technology calibrated circuit simulations to characterized the variability of the leakage time constant and the input gain. We applied a single voltage pulse of 50 ns in rise/fall time and 1μs pulse-width and monitoring the voltage at the capacitor over time. The results are repeated for 50 different circuits and the results are summarized in Supplementary Fig 2c-f.

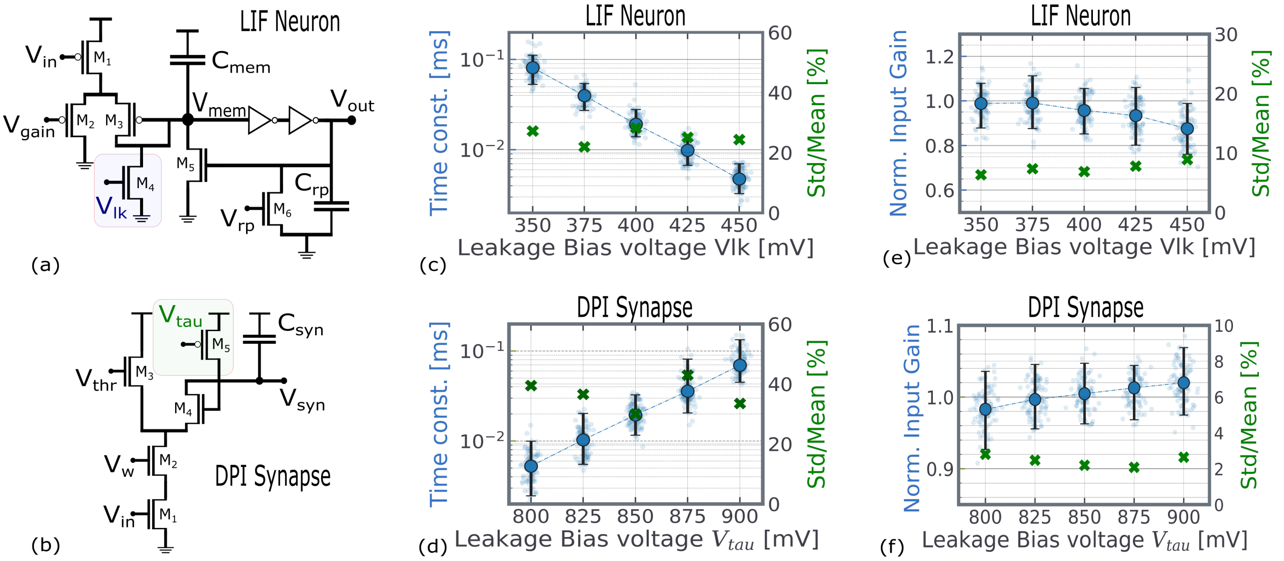

Supplementary Figure 2: Variability analysis of neuron and synapse analog circuits by means of technology calibrated circuital simulations. A single pulse is applied at the input and the response of the neurons and synapse is analyzed. (a,b) LIF neuron and DPI synapse circuit. (c,d) Both neuron and synapse allow for time constants in the range [10 μs, 500 μs], with a variability quantified around 30% (standard deviation over mean). (e,f) Input gain variability is around 8% for the neuron and 3% for the synapse.

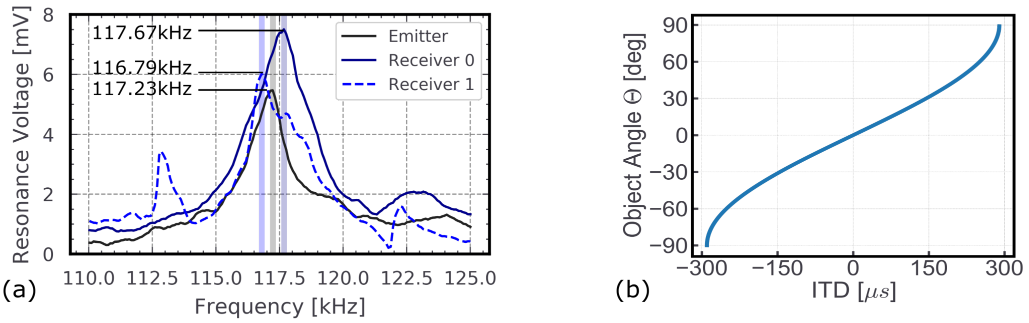

Supplementary Figure 3: PMUT characterization and Object Angle as a function of ITD. (a) Resonance voltage amplitude of the 3 pMUT membranes as a function of the input driving voltage frequency. Each membrane shows a slightly different resonance frequency, due to imperfections in fabrication. This mismatch does not affect the quality of the measurements. (b) Analytical relationship between the Interural Time Difference (ITD) and angular/azimuthal object position.

RRAM Calibration Procedure in delay line and direction insensitive coincidence detector We developed simple algorithms describing the RRAM calibration procedure in the delay line (left) and direction insensitive CD (right). The RRAMs in the circuit are repeatedly re-programmed modulating the compliance current I_{CC}, thus controlling the resulting conductance. Employing the Relaxation Correction Smart Programming (Supplementary Fig. 1e, f) one can obtain stable and precise RRAM conductance states. To speed up convergence, the modulation of the compliance current depends on the error that either the delay line or direction insensitive CD output present.

Algorithm 1 Delay Line RRAM calibration

$G_0 \leftarrow SET_{HCS}(I_{CC})$
T_{del} : target delay
$t_{del} \leftarrow$ apply input pulse
tol: tolerance for the delay
while $t_{del} < T_{del} - tol$ or $t_{del} > T_{del} + tol$:
    $t_{del} \leftarrow$ apply input pulse
    if $t_{del} > T_{del} + tol$:
        $I^{'}_{CC} = I_{CC} + \Delta I$
        $G_0 \leftarrow SET_{HCS}(I^{'}_{CC})$
        Wait 5s (Relaxation correction)
    elif $t_{del} < T_{del} - tol$:
        $I^{'}_{CC} = I_{CC} - \Delta I$
        $G_0 \leftarrow SET_{HCS}(I^{'}_{CC})$
    end
end

Algorithm 2 Direction Insensitive CD calibration

$G_0, G_1 \leftarrow SET_{HCS}(I^{'}_{CC})$
$[0, \Delta t_{CD}]$: target coincidence detector range

$CD_{response} \leftarrow$ apply input pulses
while CD does not respond to $[0, \Delta t_C D]$ only:
    $CD_{response} \leftarrow$ apply input pulses
    if CD not responding to dt in $[0, \Delta t_{CD}]$:
        $I^{'}_{CC} = I_{CC} + \Delta I$
        $G_0, G_1 \leftarrow SET_{HCS}(I^{'}_{CC})$
        Wait 5s (Relaxation correction)
    elif CD responding to dt in $dt > \Delta t_{CD}$:
        $I^{'}_{CC} = I_{CC} - \Delta I$
        $G_0, G_1 \leftarrow SET_{HCS}(I^{'}_{CC})$
    end
end

pMUT sound reception and pre-processing stage Supplementary Fig. 4 reports all the stages and corresponding hardware components of the adopted neuromorphic pre-processing technique converting the two pMUT output sound waves into spikes for the neuromorphic computational map. The same procedure is adopted for the two pMUT receivers. The reflected sound wave reaches the two pMUT receivers in a slightly different time, depending on the azimuthal position of the object. The frequency of the reflected sound wave is approximately the same as the sound wave produced by the emitter pMUT and its peak amplitude corresponds to the Time-of-Flight (ToF). The ToF is extracted from each of the two channels and encoded as the precise timing of a single spike. The pMUT raw signal is first magnified by a pre-amplifier and then smoothed by a Band-Pass filter centered around the pMUTs oscillation frequency. The resulting signal is Half-Wave rectified to remove the negative component and then fed into n Leaky-Integrate-and-Fire (LIF) neuron circuit. The threshold of the LIF is calibrated to obtain a single output spike at the ToF of the reflected sound wave detected by the pMUT receiver. Since the amplitude of the pMUT response decays as a function of the object distance, the threshold of the LIF neuron must decay over time as well: an exponentially decaying threshold correctly reproduces the behavior of the decrease in peak amplitude. An up-level-shifter ensures that the neuromorphic pre-processing and the neuromorphic computational map, operating at different voltages, operate correctly when connected together. The output spikes are then directly sent to the delay lines and coincident detector modules organized in parallel into the neuromorphic computational map. Since the spikes are sent to the gate of the RRAM assess transistors no additional amplification circuitry is required.

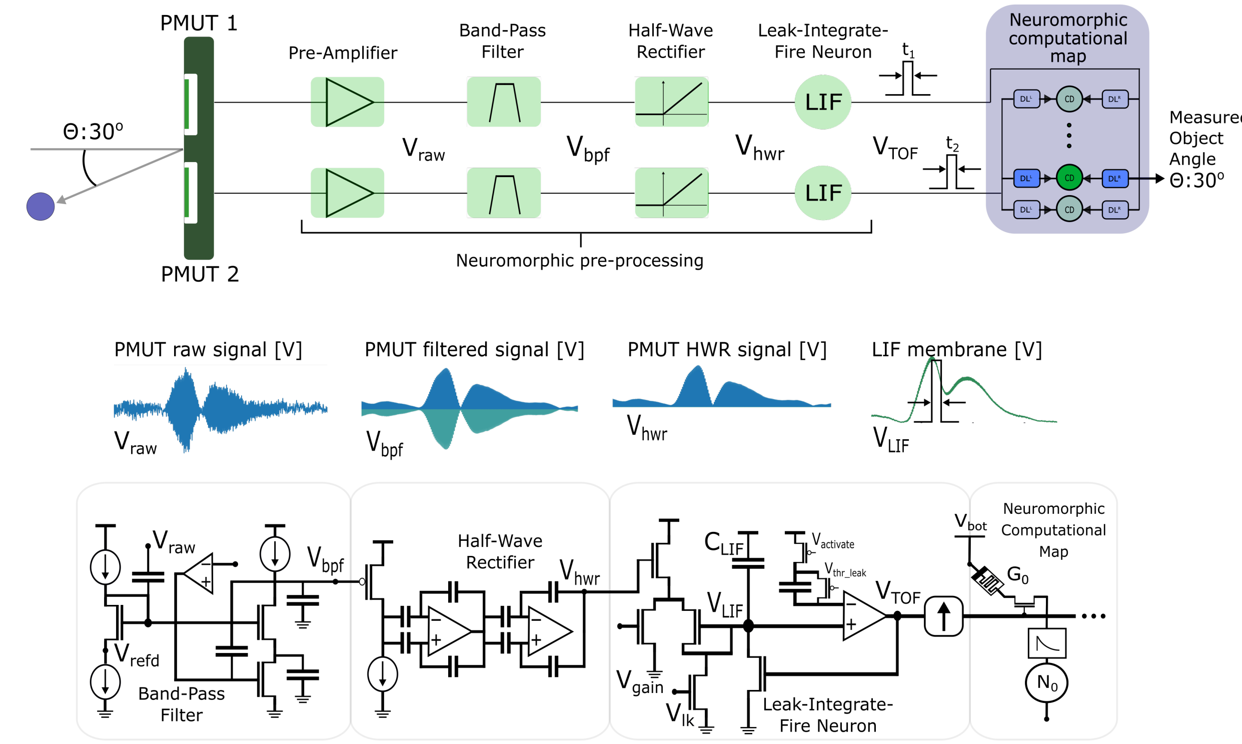

Supplementary Figure 4: PMUT data treatment assessment. From left to right, the Raw signal of the pMUT is converted to a Voltage: at this stage it is hard to retrieve any information from the signal. A Band-Pass Filter (BPF) centered around the pMUT resonance frequency is applied to remove most of the noise and smoothen the signal. Later, the BPF signal is Half-Wave-Rectified to remove the negative component. The resulting signal is feeding a Leaky-Integrate-and-Fire (LIF) neuron. This LIF neuron features an exponentially decreasing threshold which, once overcome, induces a single output spike emitted at the Time-of-Flight of the sound wave detected by the pMUT receiver. Circuits on the lower part of the figure are taken from [2].

Comparison of our memristor-based object localization system with the state-of-the-art The precision on the target object angular position, i.e. the standard deviation of the measurements, was assessed at 10°at 50 cm for the proposed 2D object localization system for a total system power consumption of 81.6 nW. Supplementary Table 1 presents a comparison with previous works, namely two pMUT-based systems for object localization [3] and rangefinding [4], and a neuromorphic memristor-based system for sound localization [5]. The 3D system leverages multiple pMUT devices (2 emitters and 7 receivers) and classical frame-based signal processing to obtain the best localization precision, at the expense of orders of magnitude more power consumption for the same measurement rate [3].

	Przybyla [2015] [3]	Chiu [2021] [4]	Jin [2014] [6]	Gao [2022] [5]	This work
Application	Object localization 3D	Rangefinding 1D	Sound localization 2D	Sound localization 2D	Object localization 2D
Processing type	Beamforming	TOF estimation	Cross-correlation	Memristor-based Neuromorphic	Memristor-based Neuromorphic
Sensory system	pMUT (2 TX, 7 RX)	pMUT (1 TX, 1 RX)	3 Microphones	2 Microphones	pMUT (1 TX, 2 RX)
Localization precision	0.2°@ 50 cm (angular)	0.63 mm @ 50 cm (distance)	1.45°@ 1 m (angular)	9.0°@ 1 m (angular)	10.0°@ 50 cm (angular)
Power consumption	1.36 mW @ 100 fps	363 μW @ 100 fps	5.63 mW	30.6 μW	81.6 nW @ 100 fps

Supplementary Table 1: Benchmarking results for the resistive memory based object localization system of this work compared to two pMUT-based systems for object localization and rangefinding, and with a neuromorphic memristor-based system for sound localization.

Supplementary References

[1] E. Esmanhotto, L. Brunet, N. Castellani, D. Bonnet, T. Dalgaty, L. Grenouillet, D. R. B. Ly, C. Cagli, C. Vizioz, N. Allouti, F. Laulagnet, O. Gully, N. Bernard-Henriques, M. Bocquet, G. Molas, P. Vivet, D. Querlioz, JM. Portal, S. Mitra, F. Andrieu, C. Fenouillet-Beranger, E. Nowak, and E. Vianello. High-density 3D monolithically integrated multiple 1T1R multi-level-cell for neural networks. In *2020 IEEE International Electron Devices Meeting (IEDM)*, pages 36.5.1–36.5.4, 2020.

[2] Minhao Yang, Hongjie Liu, Weiwei Shan, Jun Zhang, Ilya Kiselev, Sang Joon Kim, Christian Enz, and Mingoo Seok. Nanowatt acoustic inference sensing exploiting nonlinear analog feature extraction. *IEEE Journal of Solid-State Circuits*, pages 1–1, 2021.

[3] Richard J. Przybyla, Hao-Yen Tang, André Guedes, Stefon E. Shelton, David A. Horsley, and Bernhard E. Boser. 3D ultrasonic rangefinder on a chip. *IEEE Journal of Solid-State Circuits*, 50(1):320–334, 2015.

[4] Yihsiang Chiu, Chen Wang, Dan Gong, Nan Li, Shenglin Ma, and Yufeng Jin. A novel ultrasonic tof ranging system using AlN based PMUTs. *Micromachines*, 12(3), 2021.

[5] Bin Gao, Ying Zhou, Qingtian Zhang, Shuanglin Zhang, Peng Yao, Yue Xi, Qi Liu, Meiran Zhao, Wenqiang Zhang, Zhengwu Liu, Xinyi Li, Jianshi Tang, He Qian, and Huaqiang Wu. Memristor-based analogue computing for brain-inspired sound localization with in situ training. *Nature Communications*, 13, 2022.

[6] Jungdong Jin, Jin Seunghun, Lee SangJun, Kim Hyung Soon, Choi Jong Suk, Kim Munsang, and Jeon Jae Wook. Real-time sound localization using generalized cross correlation based on 0.13 μm CMOS proces. *Journal of Semiconductor Technology and Science*, 14:175–183, 2014.
